# The cardiometabolic transition in Mexico: cohort-level evidence from the ENNVIH, 2002–2009

**DOI:** 10.3389/fnut.2026.1926732

**Published:** 2026-10-30

**Authors:** Kely Rely, Israel Rico-Alba, Guillermo Salinas-Escudero, Pierre Alexandre Kebreau, María Fernanda Carrillo-Vega, Charles Joseph Fleurimonde

**Affiliations:** 1 Independent Consultant in Health Economics and Public Policy Evaluation, Mexico City, Mexico; 2 Postgraduate Studies Division, Faculty of Accounting and Administration, National Autonomous University of Mexico, Mexico City, Mexico; 3 Centre for Economic and Social Studies in Health, Federico Gómez Children’s Hospital, Mexico City, Mexico; 4 Department of Healthcare Economics, Florida Atlantic University, Boca Raton, FL, United States; 5 Research Department, National Institute of Geriatrics, Mexico City, Mexico; 6 Independent Consultant, Mexico City, Mexico

**Keywords:** abdominal obesity, BMI, Mexico, cardiometabolic transition, ENNVIH, nutritional transition, pseudo-panel

## Abstract

**Introduction:**

Mexico experienced one of the fastest cardiometabolic transitions in Latin America between 2002 and 2009, but longitudinal evidence on its underlying drivers remains limited. This study aims to characterize within-cohort changes in four cardiometabolic indicators.

**Methods:**

A strongly balanced pseudo-panel of 14 synthetic cohorts (7 age groups × 2 sexes; ages 12–74 in 2002) was constructed from three waves of the Mexican Family Life Survey (2002, 2005, and 2009), yielding 42 cohort-wave observations. Cohort fixed-effects models with robust standard errors clustered at the cohort level were estimated, using 2002 as reference year. Because official expansion factors were unavailable for the 2009 wave, estimates from this survey were derived from unweighted analyses and should be interpreted with appropriate caution.

**Results:**

Mean BMI increased by 1.23 kg/m^2^ (95% CI: 0.35–2.11; *p* = 0.010) between 2002 and 2009. Waist circumference increased more rapidly, by 8.55 cm (95% CI: 6.32–10.78; *p* < 0.001). The longitudinal waist-to-BMI relationship significantly exceeded the cross-sectional baseline, indicating that abdominal obesity progressed 2.75 times faster than expected from weight gain. Abdominal obesity rose from 49.1 to 73.0%, equivalent to 22.8 million additional adults meeting IDF criteria. By 2009, over 70% had abdominal obesity, versus only 30% classified obese by BMI. Self-reported diabetes increased by 4.0 percentage points and hypertension by 2.9 (*p* ≤ 0.001).

**Discussion:**

Mexico’s cardiometabolic transition was characterized by a rapid expansion of abdominal obesity that substantially outpaced BMI-defined weight gain. Routine measurement of waist circumference alongside BMI is warranted to improve cardiometabolic surveillance and guide prevention strategies targeting visceral adiposity, diabetes, and hypertension.

## 1 Introduction

The obesity pandemic represents one of the most significant public health challenges of the 21st century, with low- and middle-income countries facing a disproportionate and rapidly growing burden (1). In Mexico, between 2000 and 2023, the combined prevalence of overweight and obesity among adults increased from approximately 62% to over 75%, one of the most pronounced documented trajectories in the Americas (1). Obesity leads to increases in type 2 diabetes, hypertension, and cardiovascular disease, which today constitute the leading causes of premature mortality and disability in the country (2–4). A plausible explanation for this growing problem is Mexico’s nutritional transformation, which has been characterized as a rapid shift from undernutrition to metabolic disease, driven by energy-dense diets and sedentary lifestyles (5).

Beyond increasing overall weight, advanced stages of the nutritional transition are characterized by qualitative changes in body fat distribution. Diets rich in ultra-processed foods, refined carbohydrates, and sugar-sweetened beverages promote accumulation of visceral fat through hepatic *de novo* lipogenesis, visceral adipocyte hypertrophy, and impaired adipokine signaling (6). Declining physical activity further favors central fat deposition. These mechanisms operating simultaneously during the nutritional transition may cause waist circumference (WC), a good indicator for visceral adipose tissue (VAT), to increase disproportionately relative to body mass index (BMI), even when changes in overall body mass are modest. Unlike subcutaneous adipose tissue, VAT is metabolically more active, releasing greater amounts of free fatty acids and pro-inflammatory adipokines that promote insulin resistance, chronic low-grade inflammation, endothelial dysfunction, and ultimately increase the risk of type 2 diabetes, hypertension, and cardiovascular disease.

In the present study, we use the term *cardiometabolic transition* to describe the downstream epidemiological process through which the nutrition transition is translated into progressive biological and clinical changes, including abdominal obesity, diabetes, hypertension, and other cardiometabolic abnormalities. Thus, measuring rather than representing an alternative concept, the cardiometabolic transition complements Popkin’s nutrition transition framework by focusing on its measurable health consequences over time (7, 8).

This growing problem demands population screening and clinical diagnostic tools capable of identifying not only obesity *per se*, but also the cardiometabolic risk it entails. Typically used to diagnose obesity, the BMI has recently been disproved as a standard; it can both underestimate and overestimate adiposity and provide inadequate information about health at the individual level. Milanese et al. (9) demonstrated that the World Health Organization (WHO) BMI classification system misclassified weight status in over one-third of adults in a European population when validated against dual-energy X-ray absorptiometry. In addition, a growing body of literature challenges the adequacy of the BMI as a cardiometabolic surveillance instrument. In fact, evidence has shown that BMI-based diagnostics systematically underestimate the population at elevated cardiometabolic risk—particularly individuals with abdominal obesity but normal or moderate overall weight (10). WC has been described as a better indicator of abdominal adiposity because it more closely reflects VAT. Consequently, assessing WC alongside BMI provides complementary information on body fat distribution and a stronger biological basis for evaluating cardiometabolic risk during the nutritional transition. Although recent reports suggest that WC is not a robust measure of ongoing illness, it remains a better tool than BMI. Besides, in resource-limited clinical settings and population screening programs, it is economical, practical, and effective.

Although repeated cross-sectional surveys such as the National Health and Nutrition Survey (ENSANUT) provide nationally representative estimates of cardiometabolic risk factor prevalence, they sample different individuals at each wave. They therefore cannot directly quantify within-cohort (intragenerational) changes over time. On the other hand, evidence on the longitudinal dynamics of these parameters is scarce. Nonetheless, cohort-level longitudinal data can distinguish between within-generational change and compositional shifts across cross-sectional samples, thereby reflecting real longitudinal change. By constructing stable synthetic cohorts, the pseudo-panel approach enables the estimation of longitudinal cohort trajectories that complement, rather than replace, evidence from repeated cross-sectional surveillance.

The Mexican Family Life Survey (ENNVIH, in Spanish), which followed the same population during a period of rapid nutritional and cardiometabolic transition in Mexico (2002–2009), providing a unique opportunity to evaluate whether BMI trajectories are consistent with the observed dynamics of abdominal obesity accumulation over time. This period coincided with significant reforms in Mexico’s health system. Seguro Popular, introduced in 2003 and progressively expanded during the following years, substantially increased access to health services for previously uninsured populations (11). This expansion likely improved the detection and diagnosis of chronic conditions such as diabetes and hypertension, providing important context for interpreting temporal changes in self-reported cardiometabolic diseases observed during the ENNVIH follow-up period. This allows critical questions to be answered: Does abdominal obesity accumulate disproportionately relative to overall BMI? Does cardiometabolic deterioration accelerate over time?

Thus, this study aims to characterize within-cohort changes in four cardiometabolic indicators — obesity by BMI criteria, abdominal obesity, diabetes, and hypertension. We test two *a priori* hypotheses: (i) cardiometabolic deterioration is temporally concentrated, with greater within-cohort change in the 2005–2009 interval than in 2002–2005; and (ii) waist circumference increases disproportionately relative to BMI. We further characterize heterogeneity by age group and provide indicative projections to 2025.

## 2 Methods

### 2.1 Study design and data sources

This is a secondary longitudinal analysis using anonymized and de-identified datasets from the ENNVIH (12). Three waves were used for this analysis: Wave I (2002), Wave II (2005), and Wave III 2009. In brief, the ENNVIH is a nationally representative stratified multistage probability sample that covers four Mexican regions. Data from the health and anthropometry module (Book S; s_sa) included direct measurements of weight, height, waist circumference, and blood pressure obtained by trained nursing personnel from the National Institute of Public Health, along with self-reported morbidity. According to the official ENNVIH User Guide, the second survey wave followed the content, design, and structure of the first-wave questionnaires, supporting methodological continuity of the anthropometric assessment across survey rounds (12).

### 2.2 Pseudo-panel construction and cohort validity

The ENNVIH was initially designed as a longitudinal panel; however, it has structural limitations—selective attrition, replacement sampling in later waves, and inconsistencies in individual identifiers—that preclude reliable individual-level panel models (12). In this context, the pseudo-panel approach—formalized by Deaton et al. (13), as extended by Verbeek and Nijman et al. (14) and McKenzie et al. (15)—represents a methodologically appropriate strategy to cope with these limitations: by constructing synthetic cohorts stable in expectation across repeated cross-sections, it recovers the longitudinal dimension of analysis without requiring individual tracking.

Thus, cohorts were defined by sex (men/women) and base-year age group (age in 2002 = observed age minus [survey year minus 2002]), grouped into seven intervals consistent with the Mexican National Health and Nutrition Survey (ENSANUT): 12–17 (adolescence), 18–24 (early adulthood), 25–34 (young adulthood), 35–44 (early midlife), 45–54 (midlife), 55–64 (late midlife), and 65–74 years (older adulthood). The age group 75 + was excluded due (16, 17) to documented compositional instability (mean age increments of 1.3–1.5 years between waves, substantially below the expected ~3-year interval). The 14 cohorts × 3 waves yield a strongly balanced pseudo-panel of 42 observations. The mean cohort sizes exceeded 1,000 individuals per wave, well above thresholds recommended by McKenzie et al. (13). Stability was verified through mean age increments of 2.84–3.15 years (2002–2005) and 3.86–4.10 years (2005–2009; Supplementary Table S1).

### 2.3 Outcome variables

For studying the cardiometabolic transition, four cardiometabolic indicators were defined: obesity by body mass index (BMI) criteria, abdominal obesity, diabetes, and hypertension. The BMI (weight [kg]/height [m]^2^) and waist circumference (WC; cm) were treated as anthropometric measurements used to characterize temporal changes in body composition. BMI was used to estimate the prevalence of excess weight (overweight [BMI ≥ 25 kg/m^2^] and obesity [BMI ≥ 30 kg/m^2^]) according to WHO adult cut-off points (16, 17). For participants aged 12–17 years, BMI-for-age Z-scores were calculated using the WHO 2007 reference; overweight was defined as +1 SD < Z ≤ +2 SD, and obesity as Z > +2 SD (18). WC was used to determine the presence of abdominal obesity according to the International Diabetes Federation (IDF) criteria for adults ≥18 years (men >90 cm, women >80 cm) (19). Previous physician-diagnosed diabetes and hypertension were self-reported. Blood pressure results are reported only descriptively because between-wave compositional non-comparability was attributable to the fieldwork protocol. Before calculating BMI and cohort-level indicators, biologically implausible or technically invalid anthropometric values were treated as missing. All cohort means were subsequently derived from valid direct anthropometric measurements obtained within the standardized ENNVIH Book S protocol.

### 2.4 Role of behavioral covariates

Educational attainment, regular physical activity, and current smoking were deliberately excluded from the primary longitudinal fixed-effects model. Educational attainment was measured only in the 2002 wave; the cohort fixed effect μ_c implicitly controls for all time-invariant between-cohort characteristics; and smoking’s within-cohort temporal variation was not statistically significant (*p* = 0.418). Cross-sectional OLS regressions using 2002 data confirm that higher education (β = −0.31, *p* = 0.022) and higher physical activity (β = −3.84, *p* = 0.024) are inversely associated with cohort-mean BMI (model R^2^ = 0.741; Supplementary Table S2).

### 2.5 Statistical analysis

The primary model was a cohort within-estimator (fixed effects) (20).


$$\tilde{Y}\_\mathrm{ct}=\alpha +\delta \_t+\mu \_c+\varepsilon \_\mathrm{ct}$$

where Ỹ_ct is the cohort mean of the outcome, δ_t are wave fixed effects capturing aggregate temporal shifts, and μ_c is time-invariant unobserved cohort heterogeneity. Standard errors were clustered at the cohort level (21). Wave 2002 was the reference. Coefficients represent mean within-cohort changes; no individual causal interpretation is attributed.

To formally evaluate whether WC increased disproportionately relative to BMI, the longitudinal WC/BMI ratio (ΔWC/ΔBMI) was estimated using the delta method implemented through Stata’s nlcom command following a seemingly unrelated estimation (suest) framework. Because xtreg fixed-effects estimators are not compatible with suest, both models were re-estimated using ordinary least squares with explicit cohort indicator variables, an algebraically equivalent specification to the fixed-effects within estimator. The resulting joint variance–covariance matrix was used to propagate the covariance between ΔWC and ΔBMI. The longitudinal ratio was then formally compared with the cross-sectional baseline slope using testnl. Linear trend models (xtreg outcome t, fe) were also estimated, where t = (wave year − 2002)/7. Four sensitivity analyses were conducted: (i) exclusion of cohorts aged 65–74; (ii) restriction to cohort-waves with ≥80% nursing-measured anthropometrics; (iii) stratification by life stage; and (iv) pooled OLS with cohort dummies. In addition, robustness of statistical inference under the small number of clusters (G = 14) was evaluated using a Wild Cluster Bootstrap procedure with 9,999 replications (Supplementary material). All analyses were performed in Stata v.14.

The cardiometabolic outcomes were specified *a priori*. Sensitivity analyses were performed exclusively to assess the robustness of the primary findings and were not considered additional confirmatory hypothesis tests.

Indicative projections for 2015, 2020, and 2025 were generated using simple linear extrapolation of the observed ENNVIH trends between 2002 and 2009. These estimates should be interpreted as illustrative scenarios rather than formal forecasts, as their purpose is to describe the potential continuation of observed trajectories under unchanged conditions (22).

### 2.6 Ethical aspects

The original ENNVIH survey protocols were reviewed and approved by the corresponding institutional ethics committees for each survey wave. All participants provided informed consent during the original ENNVIH data collection according to the survey protocols. The present study is a secondary analysis of publicly available, fully anonymized and de-identified data. Therefore, no additional institutional review board approval was required for this secondary analysis.

## 3 Results

### 3.1 Sample characteristics and population-level trends

The pooled analytical sample comprised 86,870 individual observations (2002: *n* = 22,321; 2005: *n* = 24,157; 2009: *n* = 40,392). Table 1 presents population-level cardiometabolic indicators across the three survey waves. At baseline (2002), mean BMI was 26.60 kg/m^2^ (95% CI: 26.16–27.04)—already in the overweight category—with obesity prevalence at 24.0% and mean waist circumference at 85.17 cm (95% CI: 83.92–86.42). Abdominal obesity affected 49.1% of the sample at baseline, equivalent to approximately 30.5 million Mexican adults. Self-reported diabetes prevalence was 7.4% (95% CI: 6.7–8.1%) and hypertension 12.0% (95% CI: 11.2–12.7%). By 2009, all cardiometabolic indicators had worsened: obesity reached 30.2%, abdominal obesity 73.0% (53 million adults), and diabetes 11.4%, while mean waist circumference rose to 93.72 cm (95% CI: 92.71–94.73).

**Table 1:** Population-level cardiometabolic indicators by survey wave, ENNVIH 2002–2009.

Indicator	2002	2005	2009	Δ 2002–09	*p*-value
Anthropometric indicators					
BMI, mean (kg/m^2^)	26.6	26.9	27.8	**+1.2**	0.010
Excess weight (BMI ≥ 25 kg/m^2^), %	58.9	60.8	68.7	**+9.8**	0.031
Overweight only (25 ≤ BMI < 30 kg/m^2^), %	34.9%	36.6%	38.5%	+3.6%	0.098
Obesity (BMI ≥ 30 kg/m^2^), %	24.0	24.2	30.2	**+6.2**	0.006
Waist circumference, mean (cm)	85.2	90.2	93.7	**+8.5**	<0.001
Abdominal obesity, %	49.1	62.3	73.0	**+23.9**	<0.001
Self-reported morbidity					
Diabetes, %	7.4	8.0	11.4	**+4.0**	<0.001
Hypertension, %	12.0	10.3	14.9	**+2.9**	0.002
Behavioral covariates					
Regular physical activity, %	19.9	12.6	14.6	−5.3	0.072
Current smoking, %	20.2	12.9	18.1	−2.1	0.418

Pooled sample: *N* = 86,870 (2002: *n* = 22,321; 2005: *n* = 24,157; 2009: *n* = 40,392). Δ and p-values from cohort fixed-effects models (wave 2002 = reference). Abdominal obesity: >90 cm men, >80 cm women (International Diabetes Federation, 2006). Bold Δ values indicate *p* < 0.05.

A key contrast emerges from Table 1: by 2009, more than seven in ten Mexican adults met IDF criteria for abdominal obesity, whereas only three in ten met BMI-defined obesity criteria (30.2%). This gap—73.0% vs. 30.2%—illustrates how reliance on BMI alone substantially underestimates the population burden of cardiometabolic risk.

An equivalent within-cohort fixed-effects test showed statistically significant temporal variation for physical activity (ejercicio_prev; ENNVIH variable gh03) (Δ2005: β = −0.073, *p* < 0.001; Δ2009: β = −0.053, *p* < 0.001), whereas smoking showed no significant within-cohort variation (*p* = 0.418). The implications of this finding are discussed in the Limitations section.

### 3.2 Fixed-effects estimates: within-cohort temporal change

Table 2 presents cohort fixed-effects model estimates for all outcomes. Breusch-Pagan LM tests confirmed substantial cohort-level variance (chibar^2^ range: 31.9–39.2; all *p* < 0.001), formally validating the fixed-effects specification. Intraclass correlation coefficients (ρ) ranged from 0.865 (SBP) to 0.966 (hypertension), indicating that 86–97% of total outcome variation is attributable to stable between-cohort differences.

**Table 2:** Cohort fixed-effects model results, ENNVIH 2002–2009.

Outcome variable	Baseline 2002	Δ 2005 (SE)	Δ 2009 (SE)	*p* (2009)	R^2^ within	ρ (ICC)
Adiposity						
BMI (kg/m^2^)	26.60	+0.26 (0.21)	**+1.23 (0.41)**	0.010	0.440	0.877
Obesity (proportion)	0.240	+0.002 (0.009)	**+0.062 (0.019)**	0.006	0.515	0.911
Waist circumference (cm)	85.17	+**5.04 (0.73)**	**+8.55 (1.03)**	<0.001	0.835	0.903
Self-reported morbidity						
Diabetes (proportion)	0.074	+**0.006 (0.003)**	**+0.040 (0.009)**	<0.001	0.628	0.958
Hypertension (proportion)	0.120	−0.017 (0.006)	**+0.029 (0.008)**	0.002	0.601	0.966
Blood pressure—descriptive only						
SBP (mmHg) *	132.0	−13.4 (1.4)	**−4.9 (1.4)**	0.004	0.787	0.865
DBP (mmHg) *	79.1	−3.2 (0.6)	+0.5 (0.5)	0.286	0.742	0.882

*N* = 42 cohort-wave observations. Robust standard errors clustered at cohort level; wave 2002 = reference. ρ = intraclass correlation coefficient. Bold Δ values indicate *p* < 0.05. * SBP and DBP: descriptive estimates only, excluded from fixed-effects inference due to between-wave compositional non-comparability (see Methods).

BMI did not change significantly between 2002 and 2005 (+0.26 kg/m^2^; 95% CI: −0.18 to +0.71; *p* = 0.223) but increased significantly by +1.23 kg/m^2^ (95% CI: 0.35–2.11) between 2002 and 2009 (*p* = 0.010). This temporal asymmetry—the absence of significant change in the first interval and significant deterioration in the second—was consistent across all adiposity outcomes and supports the first hypothesis of temporally concentrated cardiometabolic deterioration.

Wild Cluster Bootstrap (WCB) inference yielded slightly more conservative *p*-values than the conventional asymptotic approach. For the 2009 estimates, the *p*-value for BMI increased from 0.010 to 0.054, while that for BMI-defined obesity increased from 0.006 to 0.050. Thus, both estimates moved to the conventional threshold of statistical significance, although their magnitude and direction remained unchanged.

### 3.3 Formal confirmation of disproportionate abdominal obesity

WC increased by +5.04 cm (95% CI: 3.47–6.61; *p* < 0.001) between 2002 and 2005 and by a further +8.55 cm (95% CI: 6.32–10.78; *p* < 0.001) by 2009, with a within-R^2^ of 0.835—the highest of any outcome model estimated. Linear trend analysis confirmed a rate of +8.42 cm per seven-year period (SE: 0.997; *p* < 0.001; within-R^2^ = 0.807). The longitudinal WC/BMI ratio was estimated using the Delta Method, yielding an estimated ratio of 6.96 cm per kg/m^2^ (95% CI: 4.03–9.88; SE = 1.49; z = 4.66; *p* < 0.001). A formal nonlinear comparison demonstrated that this ratio significantly exceeded the cross-sectional baseline slope observed in 2002 (2.53 cm per kg/m^2^; χ^2^(1) = 8.79, *p* = 0.003), confirming that abdominal obesity accumulated at a rate 2.75 times greater than overall weight gain would predict.

Diabetes increased at a rate of +4.1 percentage points per seven-year period (SE: 0.009; *p* < 0.001; within-R^2^ = 0.573), consistent with the fixed-effects wave estimate of +4.0 percentage points (pp) (95% CI: 2.2–5.8; *p* < 0.001). Hypertension exhibited a non-linear pattern: it declined between 2002 and 2005 (−1.7 pp.; 95% CI: −3.0 to −0.5; *p* = 0.012), before rising markedly between 2005 and 2009 (+4.6 pp.; *p* < 0.001), yielding a net increase of +2.9 pp. by 2009 (95% CI: 0.7–5.1; *p* = 0.002).

### 3.4 Integrated cardiometabolic transition overview

Figure 1 integrates the four cardiometabolic indicators documented in Table 2 into a single transition overview. The figure shows the concurrent increase in BMI, abdominal obesity, diabetes, and hypertension between 2002 and 2009. Abdominal obesity exhibited the steepest increase, rising from 49.1 to 73.0%, while diabetes increased from 7.4 to 11.4% and hypertension from 12.0 to 14.9%. Dashed lines extend the observed ENNVIH trajectories as indicative linear projections to 2015, 2020, and 2025. Taken together, these trajectories document a coherent and multidimensional cardiometabolic transition in which abdominal obesity emerges as the dominant and fastest-growing component of risk.

**Figure 1:**
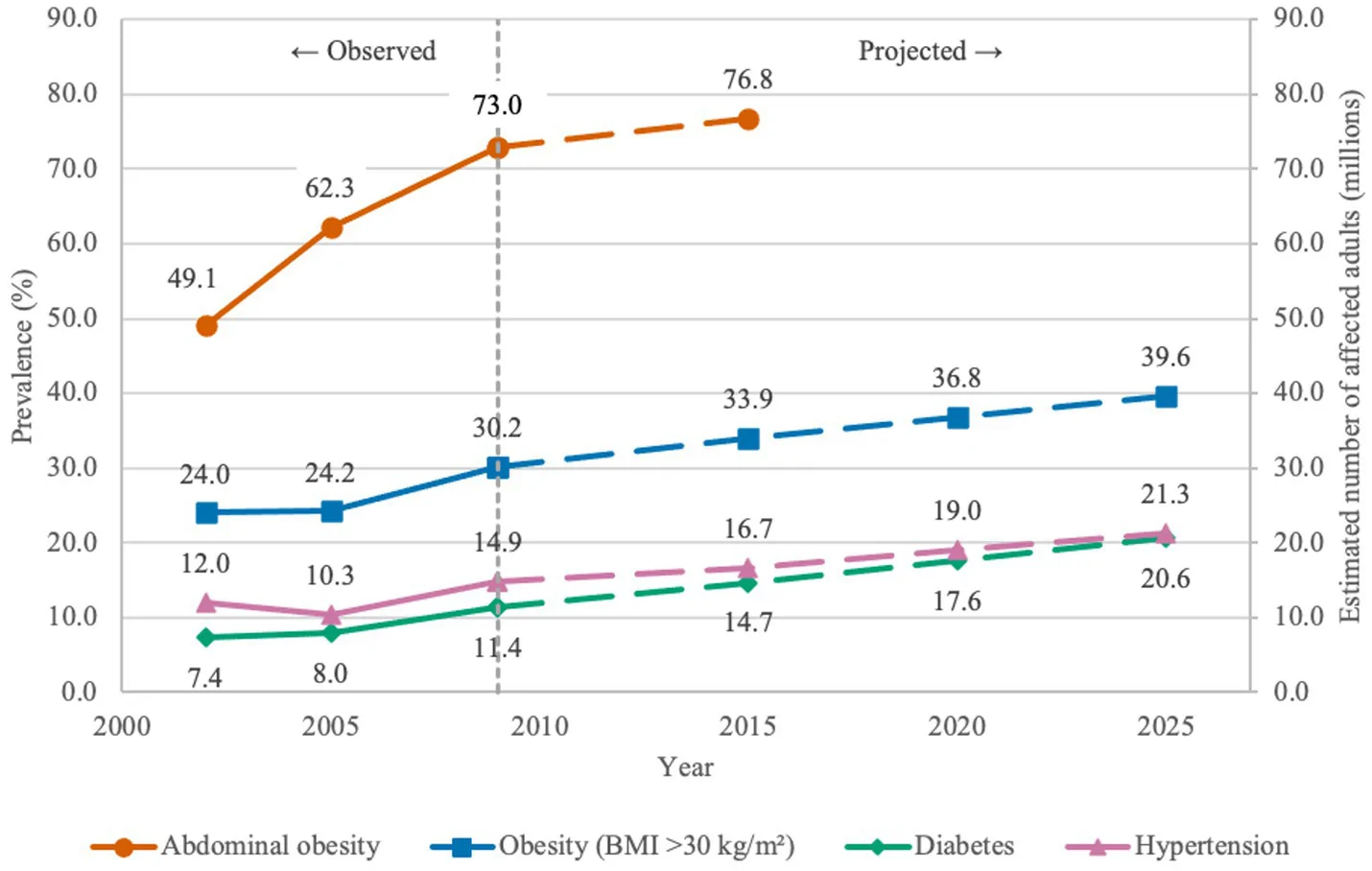
Cardiometabolic transition in Mexico: observed ENNVIH trends (2002–2009) and illustrative linear projections (2015–2025). Right axis: estimated number of affected adults (millions), calculated using official CONAPO adult population projections (ages 12–74 years), consistent with Table 5. Dashed lines represent illustrative linear projections and should not be interpreted as formal forecasts. The x-axis displays survey and projection years (2002, 2005, 2009, 2015, 2020, and 2025) at equal spacing for visualization purposes; distances between points are not proportional to the actual time intervals.

### 3.5 BMI and waist circumference trajectories by age stratum

Among younger cohorts (aged 12–54 years; Figure 2) Panel A, mean BMI increased from 26.13 to 27.96 kg/m^2^ (+1.83 kg/m^2^; *p* = 0.003), approximately 1.5 times the overall cohort average. Mean waist circumference rose from 82.69 to 92.78 cm (+10.09 cm; *p* < 0.001), crossing the IDF male threshold between the 2002 and 2005 waves. The relative increase in WC (+12.2%) substantially exceeded that of BMI (+6.6%), confirming that disproportionate central fat deposition was most pronounced in the age groups with the greatest long-term cardiometabolic implications.

**Figure 2:**
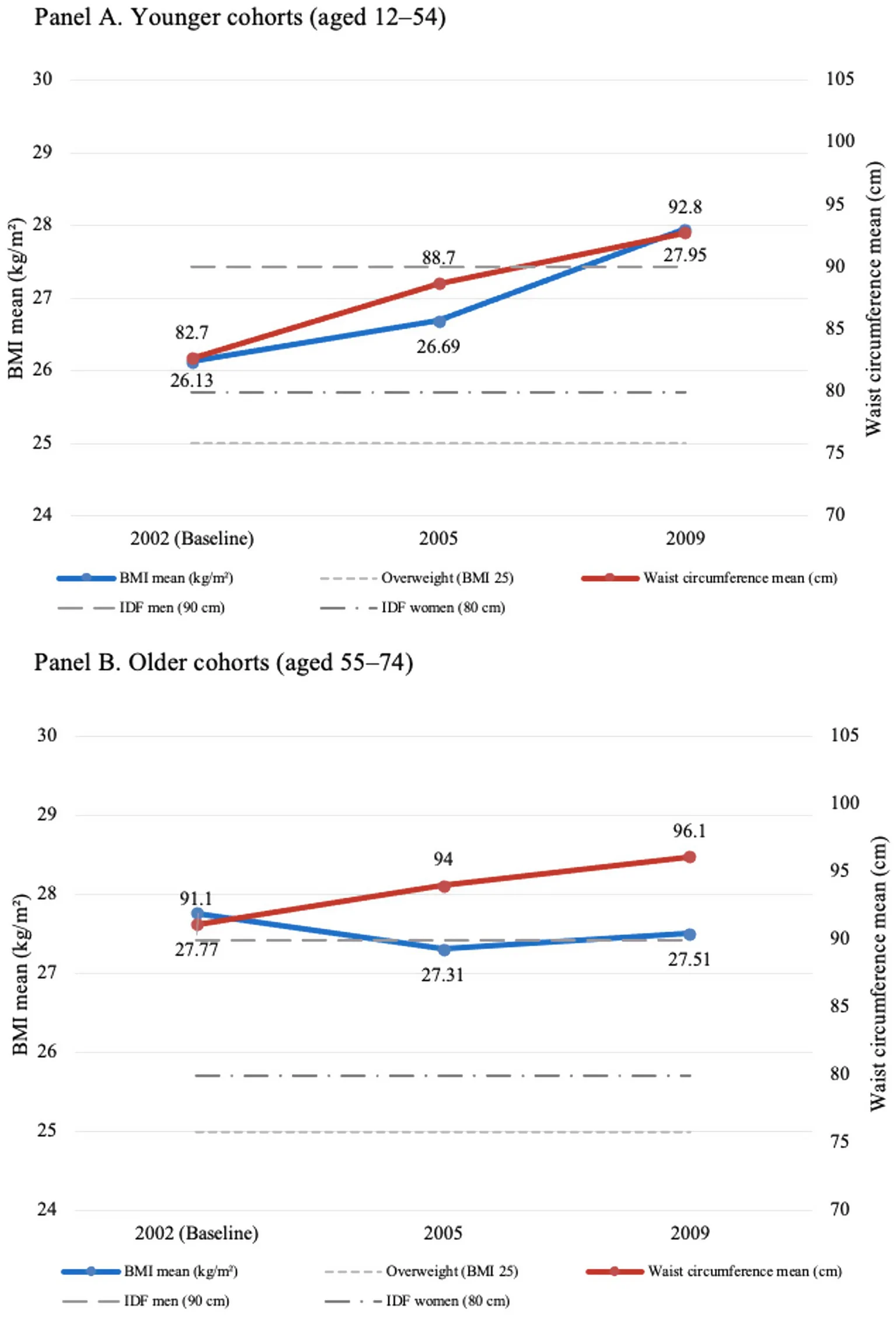
Cohort-level BMI and waist circumference trajectories stratified by age group, revealing markedly divergent patterns. Source: ENNVIH pseudo-panel (14 cohorts x 3 waves, *N* = 42 cohort-wave observations). Left axis (blue): BMI mean (kg/m^2^). Right axis (red): Waist circumference mean (cm). Dashed line: IDF (2006) abdominal obesity threshold for men (90 cm). Panel **(A)** Cohorts aged 12–54 (*n* = 30). Panel **(B)** Cohorts 55–74 (*n* = 12). Panel **(A)** Values: differences between 2009 and 2002 cohort means. All values are real data from ENNVIH. *P*-values BMI from cohort fixed-effects model (Table 2).

Among older cohorts (aged 55–74 years; Figure 2) Panel B, mean BMI remained essentially stable (27.77 → 27.31 → 27.51 kg/m^2^; Δ = −0.26 kg/m^2^; *p* = 0.058), while mean WC continued to rise from 91.36 to 96.06 cm (+4.70 cm; *p* < 0.001), already exceeding the IDF threshold at baseline.

### 3.6 Age-stratified heterogeneity

Younger cohorts (base-year ages 12–44) experienced a mean increase in BMI of +2.25 kg/m^2^ (*p* = 0.001), approximately twice the overall average. Cohorts aged 55 + exhibited BMI reductions of −0.14 to −0.39 kg/m^2^. A formal interaction test confirmed that this divergence was statistically significant: the 2009 × age55 + interaction term was −2.09 kg/m^2^ (*p* < 0.001), consistent with selective survival of leaner individuals in older age groups. Self-reported diabetes increased monotonically with age group at baseline, and the largest absolute increase (+4.0 pp) was observed across all cohorts.

### 3.7 Sensitivity analyses

Table 3 demonstrates robustness across all four alternative specifications. The pooled OLS model produced a coefficient of +1.23 kg/m^2^ (*p* = 0.002), identical to the within-estimator, confirming the absence of attenuation bias. Restricting the sample to cohort-waves with ≥80% of anthropometric measurements obtained via clinical assessment left the results unchanged, ruling out differential measurement source as a confounding factor.

**Table 3:** Sensitivity analyses: body mass index within-cohort change under alternative specifications.

Specification	Base model	Young (12–44)	Older (45–74)	Excl. 65–74	Clinical only	Pooled OLS
Δ BMI 2002–05	+0.26	+0.75 **	−0.39 *	+0.55 **	+0.26	+0.26
Δ BMI 2002–09	+1.23 **	+2.25 ***	−0.14	+1.83 ***	+1.23 **	+1.23 ***
*p*-value (2009)	0.010	0.001	0.241	0.003	0.010	0.002
R^2^ within	0.440	0.820	0.464	0.679	0.440	—
N cohorts	14	8	6	10	14	14

All models with cohort fixed effects except pooled OLS (cohort dummies included). Robust clustered standard errors. ***p < 0.01, **p < 0.05, *p < 0.10. Wave 2002 reference.

### 3.8 Indicative projections

Table 4 presents observed values with 95% confidence intervals alongside indicative linear projections to 2025. The projected diabetes prevalence is 14.7% by 2025 (95% CI: 11.0–18.3%), and the projected BMI is 30.62 kg/m^2^ (95% CI: 28.94–32.31). The hypertension projections carry wide prediction intervals (95% CI by 2025: 2.4–40.1%) owing to their non-linear trajectory and must be interpreted as illustrative estimates only.

**Table 4:** Observed values and indicative projections with 95% confidence intervals, ENNVIH 2002–2009 (observed) and 2015–2025 (projected).

Year	BMI (kg/m^2^) [95% CI] (mean)	Waist circ. (cm) [95% CI]	Diabetes (%) [95% CI]	Hypert. (%) [95% CI]
Observed values				
2002	26.60 [26.16–27.04]	85.17 [83.92–86.42]	7.4 [6.7–8.1]	12.0 [11.2–12.7]
2005	26.86 [26.78–26.95]	90.21 [89.78–90.63]	8.0 [7.5–8.5]	10.3 [9.3–11.2]
2009	27.83 [27.38–28.28]	93.72 [92.71–94.73]	11.4 [10.3–12.6]	14.9 [13.7–16.0]
Indicative projections (linear extrapolation with 95% prediction intervals)				
2015	28.83 [27.98–29.69]	101.3 [96.9–105.8]	14.7 [11.0–18.3]	16.7 [7.2–26.3]*
2020	29.73 [28.46–30.99]	107.3 [100.7–113.9]	17.6 [12.2–23.0]	19.0 [4.8–33.2]*
2025	30.62 [28.94–32.31]	113.4 [104.6–122.1]	20.6 [13.4–27.8]	21.3 [2.4–40.1]*

Observed: cohort fixed-effects model, 14 cohorts × 3 waves. Projections: linear extrapolation from OLS trend model estimated on observed cohort means. 95% CIs: lincom post-estimation (observed); OLS prediction intervals (projections). * Wide CIs due to non-linear trajectory (within-R^2^ = 0.281); interpret as illustrative estimates.

### 3.9 Population burden estimates

Table 5 translates these prevalence changes into absolute population burden. The number of Mexican adults meeting IDF criteria for abdominal obesity increased from approximately 30.5 million in 2002 to 53.3 million in 2009, representing about 22.8 million additional adults in only 7 years. Over the same period, the number of adults with self-reported diabetes increased from approximately 4.6 million to 8.3 million. These estimates show that the cardiometabolic transition was not only a relative epidemiological shift, but also a major expansion in the absolute number of people requiring prevention, screening, and long-term care.

**Table 5:** Estimated population burden of cardiometabolic risk indicators, Mexico 2002–2009 (observed) and 2015–2025 (projected).

Year	Adult population (millions) *	BMI (kg/m^2^) > 30 (millions) [%]	Waist circumference/(millions) [%]	Diabetes (millions) [%]	Hypertension (millions) [%]
Observed values					
2002	62.2	14.9 [24.0%]	30.5 [49.1%]	4.6 [7.4%]	7.5 [12.0%]
2005	67.0	16.2 [24.2%]	41.7 [62.3%]	5.4 [8.0%]	6.9 [10.3%]
2009	73.0	22.0 [30.2%]	53.3 [73.0%]	8.3 [11.4%]	10.9 [14.9%]
Indicative projections					
2015	82.0	27.8–35.8 [33.9%]	63.0 [76.8%]	9.0–15.0 [14.7%]	5.9–21.6 [16.7%] **
2020	89.0	26.4–43.9 [36.8%]	N/A ††	10.9–20.5 [17.6%]	4.3–29.6 [19.0%] **
2025	95.0	31.2–52.6 [39.6%]	N/A ††	12.7–26.4 [20.6%]	2.3–38.2 [21.3%] **

Prediction intervals for projected BMI-defined obesity, diabetes, and hypertension were estimated using the same longitudinal fixed-effects trend model to ensure methodological consistency across projected prevalence indicators. ^††^N/A. Not reported because linear extrapolation produced biologically implausible projected prevalences (>100%) beyond the valid projection horizon for abdominal obesity.

## 4 Discussion

Our study provides the first cohort-level longitudinal analysis of concurrent anthropometric and self-reported morbidity indicators in Mexican adults using the ENNVIH and makes two main contributions: an indicative projection of population-level cardiometabolic burden through 2025 and a formal statistical confirmation that abdominal obesity outpaced overall weight gain. Taken together, the four indicators (obesity by BMI criteria, abdominal obesity, diabetes, and hypertension) describe a coherent cardiometabolic risk profile. This distinction is important because repeated cross-sectional surveys, including ENSANUT, are designed to monitor population prevalence rather than within-cohort change. Consequently, they cannot directly estimate the intragenerational cardiometabolic trajectories that constitute the primary focus of the present study.

The observed 2009 ENNVIH estimates were also broadly consistent with independent national surveys conducted during the same period, supporting the external plausibility of our findings. The prevalence of obesity (BMI ≥ 30 kg/m^2^) reached 30.2% in ENNVIH, closely matching the 30.4% reported in ENSANUT 2006 and the 32.4% reported in ENSANUT 2012 (23). Similarly, abdominal obesity reached 73.0%, comparable to the 75.5% observed in ENSANUT 2006 and the 74.1% reported for ENSANUT 2018–19 using the same International Diabetes Federation criteria (1). Self-reported hypertension in ENNVIH (14.9%) was virtually identical to the national estimate reported in ENSANUT 2012 (24). Although self-reported diabetes prevalence in ENNVIH (11.4%) exceeded the estimates reported by ENSANUT 2006 (7.3%) and ENSANUT 2012 (8.8%), both surveys consistently documented an increasing burden of diabetes over time, supporting the epidemiological plausibility of the observed trend rather than suggesting contradictory findings (3). Taken together, these comparisons suggest that the ENNVIH observations are epidemiologically coherent with independent national surveillance data rather than representing isolated survey-specific findings. The consistency between ENNVIH and independent ENSANUT estimates strengthens confidence in the quality of the longitudinal measurements and supports the use of ENNVIH for investigating cardiometabolic trajectories in the Mexican population.

### 4.1 A rapid multidimensional cardiometabolic transition

Across all four cardiometabolic indicators simultaneously—BMI obesity, abdominal obesity, diabetes, and hypertension (Figure 1)—a coordinated deterioration with no single-factor explanation was observed for the 2002–2009 period. The temporal structure of this transition is itself informative: the absence of significant BMI change in the first sub-period (2002–2005) followed by significant deterioration in the second (2005–2009) is consistent with a threshold-and-acceleration dynamic rather than linear drift. The 2005–2009 interval aligns with the period of the most intense expansion in ultra-processed food availability in Mexico (25) and with the rapid urbanization and dietary globalization that characterize stage 4 of Popkin’s nutrition transition (7, 8). The concentration of cardiometabolic deterioration in this second sub-period warrants specific epidemiological monitoring.

### 4.2 Waist circumference outpaces BMI

A main finding of this study is not that BMI increased, but that abdominal obesity increased far more rapidly than BMI-defined obesity. As seen, the longitudinal WC/BMI ratio exceeded the cross-sectional baseline reference by a factor of 2.75—a margin not accessible from cross-sectional BMI comparisons—providing formal statistical evidence that abdominal obesity accumulated disproportionately. These results illustrate the scenario described by Milanese et al. (9): individuals who appear stable or improving by BMI criteria are nonetheless accumulating clinically significant visceral fat. Our findings are clinically relevant, as abdominal obesity is a stronger independent predictor of insulin resistance, metabolic syndrome, type 2 diabetes, and cardiovascular events than BMI alone (26, 27). Besides, indicative projections suggest an extremely high burden of abdominal obesity if trends continue, with a projected waist circumference by 2025 that exceeds the IDF male threshold by a wide margin, even under conservative scenarios. This pattern is biologically plausible within the framework of the nutritional transition. Experimental and epidemiological evidence indicates that diets characteristic of advanced nutritional transition stages fosters visceral rather than peripheral fat deposition, resulting in greater increases in waist circumference than would be expected from changes in BMI alone (6). Waist circumference has recently been endorsed as a clinical vital sign that should be routinely measured alongside BMI precisely because of its superior sensitivity to visceral adiposity accumulation (27). The longitudinal divergence between WC and BMI growth documented here (+8.55 cm vs. +1.23 kg/m^2^) is consistent with this biological rationale. The bounded sensitivity analyses further showed that waist circumference was the most stable indicator across alternative projection scenarios (Supplementary Figure S1), reinforcing the robustness of abdominal obesity as the predominant anthropometric manifestation of the cardiometabolic transition observed in this cohort.

### 4.3 Why BMI-based surveillance underestimates cardiometabolic risk

Our results show that individuals aged 55 + had no significant change in BMI between 2002 and 2009, yet experienced a + 4.70 cm increase in waist circumference, which was already above the IDF threshold at baseline. Moreover, by 2009, more than seven in ten adults met the criteria for abdominal obesity, compared with fewer than one in three meeting the BMI-defined obesity criteria. Previous evidence from Mexico consistently shows that a considerable proportion of adults with normal BMI already exceed IDF waist circumference thresholds, stressing the limitations of relying exclusively on BMI to identify individuals at elevated cardiometabolic risk (28, 29). These data are consistent with international evidence showing that measures of abdominal obesity provide additional prognostic information beyond BMI alone, i.e., Coutinho et al. demonstrated that combining BMI with measures of abdominal obesity improved the assessment of mortality risk, highlighting the limitations of relying exclusively on BMI as an indicator of cardiometabolic health (30).

An additional finding from the age-stratified analysis was the attenuation of BMI among the oldest cohorts despite the continued increase in waist circumference. This pattern is consistent with one of the few longitudinal cohort studies conducted in Mexico, which documented progressive reductions in BMI and transitions toward lower BMI categories among older adults during two decades of follow-up (31). These findings suggest that age-related weight loss, survival effects, and changes in body composition may partially explain the attenuation of BMI observed in later life despite the overall increase in obesity across younger age groups.

These findings support the routine incorporation of waist circumference alongside BMI in national cardiometabolic surveillance systems and clinical settings (29)—a complementary rather than a substitutive approach that would identify a substantially larger high-risk population than BMI alone.

### 4.4 Population burden and health-system implications

Expressing the findings as population burden fundamentally changes the public health interpretation. A rise in abdominal obesity from 49.1 to 73.0% is not only a statistical increase; it represents approximately 22.8 million more adults exposed to elevated cardiometabolic risk in seven years. This has direct implications for primary care, diabetes screening, hypertension detection, and long-term cardiovascular disease prevention. The number of adults with diabetes grew from 4.6 million to 8.3 million over this period, and indicative projections suggest this could reach 13–26 million by 2025. Although these projections were intended as illustrative rather than predictive, the observed prevalence estimates from independent ENSANUT surveys conducted during comparable periods remained broadly compatible with the magnitude of the ENNVIH observations, supporting the external plausibility of the longitudinal trends described here. The non-linear hypertension trajectory—a decline in 2005 followed by a sharp rebound in 2009—likely reflects a policy effect of the Seguro Popular program rather than genuine epidemiological improvement, followed by a resumption of the underlying deterioration. This pattern underscores the importance of objective anthropometric indicators alongside self-reported morbidity in tracking population health.

The post-2009 period, however, included major public-health interventions and epidemiological shocks that make a simple continuation of the 2002–2009 slope unlikely. Mexico introduced a 1-peso/L excise tax on sugar-sweetened beverages in 2014; evaluations documented sustained reductions in purchases of taxed beverages during 2014–2015 and decreases of overweigth and obesity prevalences only in certain subpopulations (32, 33). Mandatory front-of-pack warning labels for packaged foods and non-alcoholic beverages were implemented in October 2020. Modeling work suggested that this policy could reduce caloric intake and obesity prevalence (34), and subsequent empirical evidence documented reformulation toward lower levels of calories and critical nutrients in several product categories (35). These interventions could have attenuated continuation of pre-2009 dietary risk trajectories. Conversely, the COVID-19 pandemic generated an opposing health-system shock: diabetes and hypertension services were substantially disrupted during 2020 (36), and diabetes-related mortality increased markedly relative to 2017–2019 (37). Accordingly, the 2025 projections should be interpreted as counterfactual extensions of the pre-2009 trajectory rather than estimates of the observed post-2009 path.

### 4.5 Strengths and limitations

Three methodological strengths distinguish this study: (1) longitudinal cohort tracking rather than cross-sectional description, enabling within-cohort inference unavailable from national surveys like the ENSANUT; (2) explicit follow-up of waist circumference alongside BMI, including a formal statistical comparison of their longitudinal divergence (29); and (3) a broader cardiometabolic profile integrating BMI, waist circumference, abdominal obesity and self-reported morbidity indicators. The cohort fixed effects control for all time-invariant between-cohort heterogeneity, and the fixed-effects specification was formally validated by Breusch-Pagan LM tests (29). Results are robust across four sensitivity specifications. The official ENNVIH documentation indicates that anthropometric data were collected as direct measurements within the standardized Book S (Anthropometrics and Biomarkers), implemented by the National Institute of Public Health. Moreover, the official User Guide documents continuity in the content, design, and structure of the survey questionnaires across waves. Taken together, these features support the comparability of the anthropometric measurements across survey waves and reinforce the interpretation of the observed longitudinal increase in waist circumference. The Wild Cluster Bootstrap sensitivity analysis supported the overall robustness of the findings. Although the BMI coefficient remained virtually unchanged, its statistical significance became borderline after applying the conservative small-cluster correction (Supplementary Table S1). The remaining outcomes showed consistent inference under both conventional and Wild Cluster Bootstrap estimation.

Principal limitations include: the cohort-level estimates cannot be attributed to individual mechanisms. Physical activity (ENNVIH variable gh03) exhibited statistically significant within-cohort variation across survey waves. However, the complete comparability of the underlying physical activity questionnaire (gh03) across the three ENNVIH rounds could not be verified from the available documentation. Therefore, although physical activity was retained as a descriptive contextual characteristic, it was not incorporated into the primary longitudinal model. Accordingly, this should be considered a potential source of residual confounding when interpreting the longitudinal findings. Official expansion factors for the 2009 ENNVIH health module were unavailable; consequently, analyses for this survey were conducted using unweighted data. As a result, the 2009 estimates may be less representative of the national target population than those from the earlier survey waves. No formal adjustment for multiple comparisons was applied because the analyses addressed a limited set of correlated, pre-specified outcomes rather than multiple independent hypotheses. Accordingly, interpretation was based on the consistency of effect sizes, 95% confidence intervals, and findings across complementary analyses rather than on isolated *p*-values.

A further limitation is that the post-2009 projections do not explicitly model structural changes occurring between the end of ENNVIH follow-up and 2025, including the sugar-sweetened beverage tax, front-of-pack warning labeling and related product reformulation, or the COVID-19-related disruption of chronic-disease care. The direction and magnitude of these forces may differ across outcomes and periods and cannot be identified from the ENNVIH data. This post-2009 structural uncertainty strengthens the rationale for the bounded-scenario sensitivity analysis reported in the Supplementary material, which evaluates deceleration, status-quo, and reacceleration trajectories rather than relying on a single linear extrapolation.

In conclusion, Mexico’s cardiometabolic transition between 2002 and 2009 was characterized by a rapid expansion of abdominal obesity that substantially outpaced BMI-defined overall weight gain. These findings support the routine incorporation of waist circumference alongside BMI in national surveillance systems and call for prevention strategies that simultaneously address visceral adiposity, diabetes risk, hypertension detection.

Finally, although the study sponsor had no role in the study design, data analysis, interpretation of the results, manuscript preparation, or publication decision, the authors acknowledge that industry funding may be perceived as a potential source of interpretive bias. Readers should therefore interpret the policy implications in the context of the study design, the objective analytical methods employed, and the transparency of the reported results.

## 5 Conclusion

This study provides the first longitudinal cohort-level evidence from ENNVIH demonstrating that Mexico’s cardiometabolic transition between 2002 and 2009 was characterized by a disproportionate increase in abdominal obesity relative to overall weight gain. By jointly evaluating the four predefined cardiometabolic indicators— obesity by BMI criteria, abdominal obesity by waist circumference, diabetes, and hypertension, our findings show that reliance on BMI alone substantially underestimates the evolving burden of cardiometabolic risk. These results support the routine incorporation of waist circumference into national surveillance systems and clinical assessment, while emphasizing the need for integrated prevention strategies targeting visceral adiposity before progression to cardiovascular disease and diabetes. More broadly, the study illustrates the value of longitudinal cohort analyses for understanding population cardiometabolic transitions beyond what can be inferred from repeated cross-sectional surveys.

## Data Availability

Publicly available datasets were analyzed in this study. This data can be found at: https://www.ennvih-mxfls.org/.
