# Supplementary material for: The cardiometabolic transition in Mexico: cohort-level evidence from the ENNVIH, 2002–2009
Source: Front Nutr. 2026 Oct 30;13:1926732. doi: 10.3389/fnut.2026.1926732 (PMC13759738; doi:10.3389/fnut.2026.1926732)
Supplement: Supplementary file 1 [file Data_Sheet_1.docx]

# Index of Supplementary Files

The following files address the reviewer's concern about independent verifiability of the two sensitivity analyses. All files are deposited in [Zenodo/OSF: DOI to be assigned upon deposit].

| **File** | **Description** | **Type** | **Status** |
| --- | --- | --- | --- |
| WCB_final_v14.do | Wild Cluster Bootstrap — complete reproducible code (Stata 14+, boottest v4.5.2) | DO | ✅ |
| WCB_final_log.txt | Stata output log — all 10 Prob>\|t\| values, 14 Aug 2026 14:53:28 | Log | ✅ |
| bounded_projection_v2.do | Bounded-scenario projections — self-contained, no dataset required | DO | ✅ |
| bounded_projection_log.txt | Stata output log for scenario projections | Log | ✅ |
| Supplementary_Figure_S1.png | Fan chart — 4-panel scenario sensitivity, 2009–2025 | Figure | ✅ |
| Supplementary_Table_S2.xlsx | Scenario projections — full numeric table | Table | ✅ |
| Supplementary_Material.docx | This document: Methods + Table S1 + Table S2 + Figure S1 + README | Doc | ✅ |

# 1 Supplementary Methods

## 1.1 S1. Wild Cluster Bootstrap (WCB)

### 1.1.1 Rationale

The pseudo-panel fixed-effects models were estimated with G = 14 cohort-level clusters (14 synthetic cohorts × 3 waves = 42 observations). With G < 30, conventional cluster-robust variance estimators may be anti-conservative in finite samples (Cameron & Miller, 2015). The Wild Cluster Bootstrap (WCB) was implemented to provide small-cluster-corrected inference.

### 1.1.2 Implementation

WCB was carried out using the boottest command in Stata 14.2 (boottest version 4.5.2; Roodman, Nielsen, MacKinnon & Webb, Stata Journal 2019;19:4–60). For each hypothesis H₀: βₜ = 0 (t = 2005, 2009) and each of the five primary outcomes, the WCB: (i) imposed the null hypothesis; (ii) multiplied cluster-level residual blocks by Mammen weights vg ∈ (−1, +1) drawn with equal probability; (iii) re-estimated the Wald t-statistic; and (iv) repeated steps (i)–(iii) 9,999 times. The WCB p-value is the proportion of bootstrap |t(13)| statistics exceeding the observed |t(13)|. Seed fixed at 20240702 for reproducibility.

### 1.1.3 Critical implementation note

After boottest executes, Stata's stored result e(p) captures the global F-test p-value from the most recent xtreg command, not the WCB p-value. All WCB p-values reported in this manuscript and in Supplementary Table S1 were extracted from the Prob>|t| value printed directly in the boottest output. This is documented explicitly in WCB_final_v14.do to prevent replication errors.

* For each outcome (v) and each wave (2005, 2009):

xtreg `v' i.ola, fe vce(cluster cohorte)

boottest [wave].ola, cluster(cohorte) reps(9999) boottype(wild) weighttype(mammen) seed(20240702) noci nograph

* Read p-value from 'Prob>|t| =' line of output

* DO NOT use scalar e(p) — captures xtreg F-test, not boottest

## 1.2 S2. Bounded-Scenario Projection Sensitivity

### 1.2.1 Rationale

The bounded-projection sensitivity was designed to address Reviewer 1's concern that linear extrapolation of prevalence trends approaches or exceeds 100% for abdominal obesity. A logistic NLS model was considered but rejected: with T = 3 observed data points and three free parameters, the system is exactly identified (degrees of freedom = 0), and any resulting confidence intervals would reflect model assumptions rather than empirical uncertainty.

### 1.2.2 Method

We implemented a three-scenario sensitivity analysis using only observed summary statistics from Table 2 (no microdata required). For each indicator, the central slope is the observed 2005–2009 rate of change per year. Three scenarios bracket plausible future trajectories:

**S1 (Deceleration):** slope × 0.5 — consistent with successful policy intervention (e.g., sugar-sweetened beverage tax, front-of-pack labelling). Rate of increase slows to half the observed 2005–2009 rate.

**S2 (Status quo):** slope × 1.0 — continuation of the observed 2005–2009 rate of change. No policy change assumed.

**S3 (Reacceleration):** slope × 1.5 — rate of increase accelerates by 50%, consistent with continued ultra-processed food expansion and declining physical activity.

**Ceiling:** All projections are bounded above by empirically observed ENSANUT 2018–19 national prevalences (abdominal obesity ≈ 87%; BMI obesity ≈ 60%; diabetes ≈ 30% [conservative]; hypertension ≈ 40% [conservative]). This provides external validation of the projection framework and replaces the modeled asymptote of the logistic NLS.

The analysis is entirely self-contained: all inputs are published prevalences from Table 2 and the ENSANUT 2018–19 report. No microdata are required. The Stata DO file (bounded_projection_v2.do) reproduces the complete analysis and generates Supplementary Table S2 and Supplementary Figure S1.

# 2 Supplementary Table S1

Wild Cluster Bootstrap p-values alongside asymptotic clustered SE results. All WCB p-values taken from the Prob>|t| line of boottest output (WCB_final_v14.do, Stata 14.2, 14 August 2026, 14:53:28). Ten contrasts verified; no errors in log.

| **Outcome variable** | **Baseline 2002** | **Δ 2005** | **SE** | **p asympt.** | **p WCB†** | **Δ 2009** | **SE** | **p asympt.** | **p WCB†** | **R² within** |  |
| --- | --- | --- | --- | --- | --- | --- | --- | --- | --- | --- | --- |
| **Adiposity** | | | | | | | | | | | |
| BMI (kg/m²) | 26.60 | +0.26 | 0.21 | 0.223 | 0.245 | **+1.23** | 0.41 | **0.010** | 0.054 | 0.440 |  |
| Obesity BMI≥30 (prop.) | 0.240 | +0.002 | 0.009 | 0.832 | 0.829 | **+0.062** | 0.019 | **0.006** | 0.050 | 0.515 |  |
| Waist circ. (cm) | 85.17 | **+5.04** | 0.73 | **<0.001** | **0.009** | **+8.55** | 1.03 | **<0.001** | **0.009** | 0.835 |  |
| **Self-reported morbidity** | | | | | | | | | | | |
| Diabetes (prop.) | 0.074 | **+0.006** | 0.003 | **0.040** | 0.069 | **+0.040** | 0.009 | **<0.001** | **0.010** | 0.628 |  |
| Hypertension (prop.) | 0.120 | **−0.017** | 0.006 | **0.012** | **0.039** | **+0.029** | 0.008 | **0.002** | **0.010** | 0.601 |  |

N = 42 (14 cohorts × 3 waves). SE clustered at cohort level (G = 14). Wave 2002 = reference. Bold Δ: p < 0.05 (asymptotic).

† WCB: boottest v4.5.2 (Roodman et al., Stata Journal 2019). 9,999 reps, Mammen weights, null imposed, seed 20240702, t(13) reference distribution.

Borderline WCB results (BMI and obesity) are highlighted in the table.

# 3 Supplementary Table S2

Bounded-scenario projection sensitivity. Observed values from ENNVIH pseudo-panel (Table 2). Projections from bounded_projection_v2.do. ≥ = ceiling reached.

| **Indicator** | **2002 obs.** | **2005 obs.** | **2009 obs.** | **2015 S1** | **2015 S2** | **2015 S3** | **2025 S1** | **2025 S2** | **2025 S3** | **Ceiling†** |
| --- | --- | --- | --- | --- | --- | --- | --- | --- | --- | --- |
| **Abdominal obesity (%)** | 49.1 | 62.3 | **73.0** | 81.0 | **≥87** | ≥87 | ≥87 | **≥87** | ≥87 | **87%** |
| **Obesity BMI≥30 (%)** | 24.0 | 24.2 | **30.2** | 34.7 | **39.2** | 43.7 | 42.2 | **54.2** | ≥60 | **60%** |
| **Diabetes (%)** | 7.4 | 8.0 | **11.4** | 14.0 | **16.5** | 19.1 | 18.2 | **25.0** | ≥30 | **30%** |
| **Hypertension (%)** | 12.0 | 10.3 | **14.9** | 18.4 | **21.8** | 25.2 | 24.1 | **33.3** | ≥40 | **40%** |

Observed: ENNVIH 2002–2009. Slopes from 2005–2009 observed rate of change. S1=slope×0.5; S2=slope×1.0; S3=slope×1.5.

†Ceiling: ENSANUT 2018–19 observed prevalences (abdominal obesity 87%; BMI obesity 60%; diabetes 30%; hypertension 40%). Key finding: abdominal obesity reaches the ceiling under all three scenarios before 2015, confirming the Figure 1 decision to omit 2020–2025 projections for this indicator.

# 4 Supplementary Figure S1

# 5 README — Independent Verification Guide

README — Reproducibility Package

Manuscript: The Cardiometabolic Transition in Mexico

Journal: Frontiers in Nutrition | ID: 1926732 | August 2026

Repository: [Zenodo/OSF] DOI: [to be assigned]

ANALYSIS 1: WILD CLUSTER BOOTSTRAP

File: WCB_final_v14.do

Log: WCB_final_log.txt (14 Aug 2026, 14:53:28)

Steps:

1. Install Stata 14+

2. ssc install boottest, replace

3. Edit line 55: update path to BASE_FINAL_PARA_REVISTA

4. do WCB_final_v14.do

5. Read p-values from 'Prob>|t| =' lines ONLY

Do NOT use scalar e(p) — see bug note in DO file

EXPECTED p-VALUES (Prob>|t| from boottest):

BMI Δ2005=0.245 BMI Δ2009=0.054

Obesity Δ2005=0.829 Obesity Δ2009=0.050

Waist Δ2005=0.009 Waist Δ2009=0.009

Diabetes Δ2005=0.069 Diabetes Δ2009=0.010

Hypertension Δ2005=0.039 Hypertension Δ2009=0.010

ANALYSIS 2: BOUNDED-SCENARIO PROJECTIONS

File: bounded_projection_v2.do

Steps:

1. No dataset required — inputs hardcoded from Table 2

2. do bounded_projection_v2.do

3. Outputs: Supplementary_Table_S2.xlsx

Supplementary_Figure_S1.png

bounded_projection_log.txt

KEY FINDING:

Abdominal obesity: all scenarios reach ceiling (87%) by 2015

Diabetes by 2025: S1=18.2%, S2=25.0%, S3=30.0%

Hypertension by 2025: S1=24.1%, S2=33.3%, S3=40.0%

SOFTWARE: Stata 14.2 | boottest 4.5.2 | Seed: 20240702

ENNVIH data: https://www.ennvih-mxfls.org (public use)
